# Mast cell degranulation and vascular endothelial growth factor expression in mouse skin following ionizing irradiation

**DOI:** 10.1093/jrr/rrab067

**Published:** 2021-08-05

**Authors:** Chang Geol Lee, Sun Rock Moon, Mee Yon Cho, Kyung Ran Park

**Affiliations:** 1 Department of Radiation Oncology, Yonsei Cancer Center, Yonsei University College of Medicine, Seoul, South Korea; 2 Department of Radiation Oncology, Wonkwang University College of Medicine, Iksan, South Korea; 3 Department of Pathology, Yonsei University Wonju College of Medicine, Wonju, South Korea; 4 Department of Radiation Oncology, Kosin University College of Medicine, Busan, South Korea

## Abstract

The present study aimed to identify the mechanisms underlying the increase in vascular permeability in mouse skin following irradiation. The left ears of C3H mice were subjected to 2 and 15 Gy of radiation in a single exposure. At 24 h after irradiation, the ears were excised and tissue sections were stained with toluidine blue to assess mast cell degranulation. Vascular endothelial growth factor (VEGF) expression was assessed via immunohistochemistry and western blotting. Approximately 5% (3%–14%) (mean [95% CI]) of mast cells in the skin of control mice were degranulated; moreover, at 24 h after 2 Gy irradiation, this value increased to approximately 20% (17%–28%). Mast cell degranulation by 15 Gy irradiation (32% [24%–40%]) was greater than that by 2 Gy irradiation. Significant differences were observed in mast cell degranulation among the control, 2 Gy and 15 Gy groups (*p* = 0.012). Furthermore, VEGF-positive reactions were observed in the cytoplasm of scattered fibroblasts in the dermis. In immunohistochemistry tests, VEGF expression at 24 h after irradiation increased slightly in the 2 Gy group compared to that in the control group, whereas no difference in VEGF expression was observed in the 15 Gy group compared to that in the control group. Expression of VEGF in western blots was consistent with that in immunohistochemistry.

In conclusion, mast cell degranulation was increased in mouse skin at 24 h after irradiation in a dose-dependent manner. In contrast, VEGF expression was slightly increased following only low-dose (2 Gy) irradiation.

## INTRODUCTION

Induction of vascular hyperpermeability is an early vascular response to ionizing radiation [1–6]; this may further contribute to subsequent fibrosis and tissue injury. The main effect of irradiation includes damage to the microvascular endothelium, thereby inducing an increase in the transvascular permeability to plasma proteins; this leads to edema and fibrin deposition in the interstitial space and blood vessel walls. The newly deposited fibrin is eventually replaced by collagen fibers, resulting in fibrosis and parenchymal cell atrophy that are characteristic of the late phase of cell injury by radiation.

Vascular permeability increases within minutes or hours after exposure to ionizing radiation [1–3]. Several studies have demonstrated that vascular permeability increases immediately after irradiation with doses as low as 2 Gy [2–4]. Both the degree and duration of hyperpermeability following irradiation are generally dependent on the radiation dose [3, 4]; however, regardless of the dose applied, a peak increase in vascular permeability is observed at 18–24 h following irradiation [1, 4–6].

One of the possible mechanisms underlying the early increase in vascular permeability following irradiation is mast cell degranulation. Mast cells play a predominant role in increasing vascular permeability following irradiation of the skin of C3H mice [4]. A peak increase in vascular permeability occurred at 24 h following 2, 15 or 50 Gy irradiation; moreover, the number of extensively degranulated mast cells significantly increased at 24 h following 15 Gy irradiation. Nevertheless, in mast cell-deficient mice, no noticeable increase was observed in the vascular permeability following irradiation. Notably, histamine released from mast cell degranulation is involved in this increased vascular permeability following irradiation [7–11].

Vascular endothelial growth factor (VEGF) is one of the most potent agents that increase vascular permeability, with 50 000 times more potency than histamine [12, 13]. VEGF protein or mRNA expression is induced at several time points following irradiation with varying doses of radiation in numerous tumors or normal cells *in vivo* or *in vitro* [14–18]. However, the protein and mRNA expression of VEGF was not increased in the normal mouse skin at 24 h following 15 Gy radiation, particularly when a peak increase was observed in vascular permeability [4]. Furthermore, it may not be possible to detect changes in VEGF levels using western blotting if the changes occur in only specific cells that compose a relatively small fraction of the tissue. Immunohistochemistry can detect such regional changes in small fractions of the tissue.

In this study, we analyzed mast cell degranulation and VEGF expression in mouse skin using three groups of mice: control group, the group that received 2 Gy and the group that received 15 Gy of radiation. Analysis was performed 24 h after irradiation, when the vascular permeability attained a peak value, to assess (i) the contribution of mast cell degranulation and VEGF to radiation-induced vascular hyperpermeability, and (ii) radiation dose–response for mast cell degranulation and VEGF expression.

## MATERIALS AND METHODS

### Animals and ear irradiation

Eight-week-old, male C3H mice were used. A single dose of 2 and 15 Gy was administered to the left ear covered with bolus of 1.5 cm thickness using 6 MV X-ray, with 100 cm source to skin distance. Four mice were used for each experiment group. The mice were intraperitoneally anesthetized with Zoletile 30 mg/kg.

### Mast cell degranulation

#### Toluidine blue staining

Ears were excised 24 h after irradiation, fixed in 10% formalin and embedded in paraffin. Thereafter, the sections were deparaffinized and hydrated in distilled water. Next, they were stained with toluidine blue solution for 2–3 min and washed three times with distilled water. Afterward, the tissue sections were rapidly dehydrated in 95% alcohol and two changes of 100% alcohol (10 dips each as the stain fades quickly in alcohol) and then cleared in xylene. Toluidine blue stains the mast cells red–purple (metachromatic staining) and the background blue (orthochromatic staining). The sections were examined at ×400 by an observer who was unaware of the identity of the individual specimens. The numbers of normal and degranulated mast cells per 10 high power field were enumerated, and the percentage of moderately and extensively degranulated cells was calculated (number of degranulated mast cell/total number of mast cell × 100).

### VEGF expression

#### Immunohistochemistry

The paraffin embedded sections were deparaffinized, rehydrated and treated with 3% hydrogen peroxide in methanol for 10 min to block the endogenous peroxidase activity and then washed with distilled water for 10 min. Thereafter, the sections were reacted with monoclonal primary antibody against VEGF (Pharmingen, San Diego, CA, USA) diluted at 1:50 for 1 h at room temperature and then washed with Tris buffer to remove the unbound antibody. Next, the sections were reacted with horseradish peroxidase polymer secondary antibody for 30 min and washed with Tris buffer for 10 min. After reacting in 3-amino-9 ethylcarbazole for 5 min, the sections were washed with running water, counterstained with hematoxylin and mounted with balsam. The primary antibody was used as a negative control.

The number of distinct positive cells was enumerated in a high-power field (×400) and was grouped as (−) for 0–4, (+) for 5–10 and (++) for >11, according to the average number of positive cells per high-power field.

#### Western blot analysis

The skin tissue samples were homogenized in lysis buffer (0.1 mol/l NaCl, 0.01 mol/l Tris–HCl, pH 7.5, 1 mmol/l EDTA and 1 μg/ml aprotinin). Thereafter, the homogenates were centrifuged at 7000 × *g* for 15 min at 4°C. The protein concentrations in the supernatants were determined by comparing a known concentration of bovine serum albumin with a commercial kit (Micro BCA, Pierce). Sodium dodecyl sulfate (SDS)-gel electrophoresis was performed in 10% polyacrylamide gel under nonreducing conditions. Lysates equivalent to 40 μg of protein from each sample were run on the gel for 90 min at 20 mA, together with a size marker (rainbow colored protein, Amersham Life Science Inc, Cleveland, OH). The electrophoresis running buffer comprised 25 mmol/l Tris base, 250 mmol/l glycine and 0.1% SDS. The proteins on the gel were subsequently transferred to a polyvinylidene fluoride transfer membrane (Micron Separations Inc) in a buffer containing 20% methanol, 39 mmol/l glycine, 48 mmol/l Tris base and 0.4% SDS.

### Statistical analysis

The Kruskal–Wallis test was used to compare the differences in mast cell degranulation among three groups. After performing the Kruskal–Wallis test, the Bonferroni correction method was applied to compare the differences between groups.

## RESULTS

### Mast cell degranulation

**Fig. 1:**
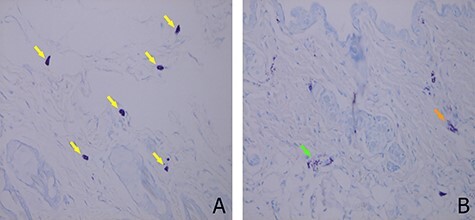
Mast cell degranulation observed in the ear skin of C3H mice by toluidine blue staining. (A) Yellow arrows indicate normal mast cell staining. (B) Orange arrow indicates moderate degranulation of mast cell, and green arrow indicates extensive degranulation of mast cell (×400).

**Fig. 2:**
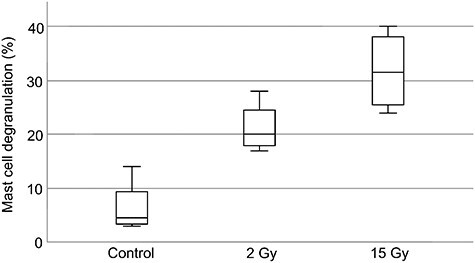
Percentage of moderate and extensive mast cell degranulation observed (mean ± 95% CI) in the ear skin of C3H mice by toluidine blue staining.

Figure 1 illustrates the mouse ear skin sections stained with toluidine blue for mast cells, and Fig. 2 depicts the percentage of mast cells with moderate and extensive degranulation features. Approximately 5% (3%–14%) (mean [95% CI]) of mast cells in the skin of control mice were initially degranulated and at 24 h after 2 Gy irradiation, this value increased to approximately 20% (17%–28%). Mast cell degranulation by 15 Gy irradiation (32% [24%–40%]) was greater than that by 2 Gy irradiation. Moreover, significant differences were observed in mast cell degranulation among the control, 2 Gy and 15 Gy groups (*p* = 0.012). The Bonferroni correction method was used to determine the differences among the groups; however, no significant difference was observed.

**Table 1:** Statistical analysis using the Kruskal–Wallis test and Bonferroni correction method for percentage of moderate and extensive mast cell degranulation observed in the ear skin of C3H mice by toluidine blue staining

	Group	N	Mean	SD	χ^2^	^*^Bonferroni correction		
						Control vs 2 Gy	2 vs 15 Gy	Control vs 15 Gy
Mast cell degranulation (%)	control	4	0.066	0.050	8.769 (p = 0.012)	n.s (p = 0.021)	n.s (p = 0.083)	n.s (p = 0.021)
	2Gy	4	0.214	0.049				
	15Gy	4	0.305	0.062				

N: Number of mice, SD: Standard deviation, n.s.: not significant, χ^2^: Kruskal–Wallis test, ^*^When comparing each two groups by the Bonferroni correction method, there is statistical significance when the p value is <0.0167.

### VEGF expression

**Fig. 3:**
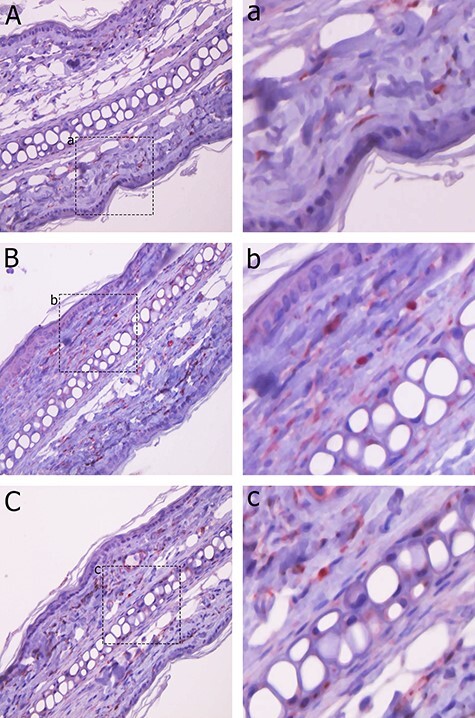
VEGF expression was observed in the ear skin of C3H mice using immunohistochemistry. VEGF expression was found in scattered fibroblasts in the dermis. A: control group. B: 2 Gy group. C: 15 Gy group. a, b and c: Enlarged image of positive cells for A, B and C, respectively. The number of VEGF-positive cells at 24 h following 2 Gy irradiation (++) slightly increased compared to that in the control group (+); however, the expression of VEGF in the 15 Gy (+) irradiation group revealed no difference compared to that in the control group (+).

**Fig. 4:**
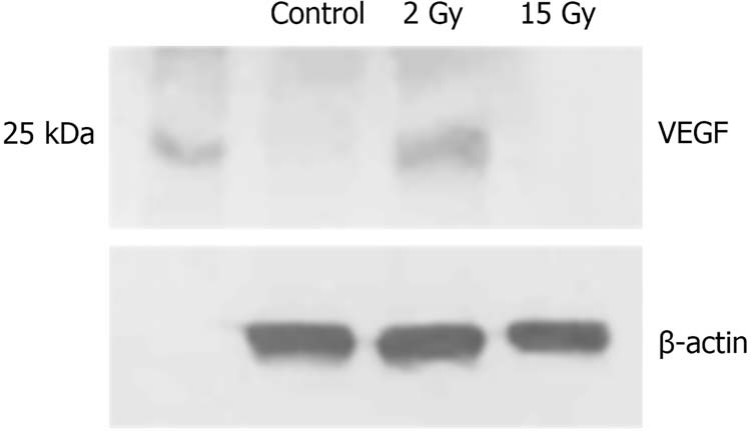
VEGF expression was observed in the ear skin of C3H mice using western blot. The expression of VEGF at 24 h following 2 Gy irradiation slightly increased compared to that in the control group; however, there was no difference between the 15 Gy group and the control group.

Immunohistochemistry revealed VEGF-positive reactions in the dermal scattered fibroblasts (Fig. 3). The number of VEGF-positive cells at 24 h following 2 Gy irradiation (++) was slightly increased compared to that in the control group (+); however, no difference was observed between the 15 Gy group (+) and the control group (+). Expression of VEGF in western blots was consistent with that in immunohistochemistry (Fig. 4).

## DISCUSSION

Endothelial cell injury is the key event in most tissues following irradiation, eventually leading to fibrosis or necrosis. The primary functional parameter related to the capillary endothelium is its permeability. Vascular permeability increases within minutes or hours after exposure to ionizing radiation [1–3] and attains a peak value at 18–24 h post irradiation [1, 4, 5, 19, 20].

Vascular permeability in the skin of C3H mice increased following 2, 15 or 50 Gy irradiation, peaked at 24 h after irradiation, and gradually decreased thereafter to the baseline level within 3–10 days. Both the extent and duration of vascular hyperpermeability were dose-dependent [4].

We analyzed mast cell degranulation and VEGF expression at 24 h following irradiation to determine the contribution of these parameters to radiation-induced vascular hyperpermeability. Our findings were in accordance with a previous study revealing that degranulation of mast cells contributed to vascular hyperpermeability [4]. The number of degranulated mast cells was significantly increased in the irradiated group at 24 h after irradiation compared to that in the control group. Moreover, the increase in degranulated mast cells after 15 Gy irradiation was greater than that after 2 Gy irradiation. Significant differences were observed in mast cell degranulation among the control, 2 Gy and 15 Gy irradiation groups (*p* = 0.012). Mast cell degranulation resulted in a dose-dependent effect.

Multiple cytokines with the ability to increase vascular permeability (e.g. histamine, serotonin and VEGF) are normally stored in tissue mast cells, which are then released by mast cell degranulation. Among various such agents, histamine potentially induces an increase in vascular permeability following irradiation [7–11]. Quantitative analysis of histamine following irradiation will help in clarifying the dose effects of radiation on mast cell degranulation.

VEGF is a potent vascular permeabilizing agent [12, 13, 21]. Mast cells produce, store and release VEGF [22]. In tumors, stromal fibroblasts and cancer cells are a significant source of VEGF that promotes angiogenesis; furthermore, fibroblasts in normal tissues also produce VEGF [23]. Several studies have shown that radiation upregulated VEGF expression at various time points, from 1 h to 20 weeks, following various doses of irradiation (0.1–75 Gy) in numerous normal tissues and tumors *in vivo* and *in vitro* [14–18]. In a previous study, northern and western blotting were used to show that the expression of VEGF protein and mRNA levels did not increase at 24 h following 15 Gy radiation in mouse skin [4].

We conducted immunohistochemistry in addition to western blotting to assess the involvement of VEGF in vascular hyperpermeability following irradiation by detecting local changes in VEGF expression in small fractions of the tissue. Immunohistochemical staining revealed that VEGF-positive reactions were observed in the cytoplasm of dermal fibroblasts. Both immunohistochemistry and western blot results indicated that at 24 h after low-dose (2 Gy) irradiation, the expression of VEGF was slightly greater in the 2 Gy irradiation group than in the control group; however, the expression of VEGF in the high-dose (15 Gy) group did not differ from that in the control group. These results indicate that VEGF was not involved in vascular hyperpermeability at 24 h following irradiation.

In conclusion, our study corroborates the results of previous studies that degranulation of mast cells contributes to vascular hyperpermeability at 24 h following irradiation, whereas VEGF does not cause hyperpermeability. Further studies are needed to reveal the radiation-induced mast cell degranulation and VEGF expression at different times after irradiation and determine the molecular mechanism of mast cell degranulation induced by radiation to develop novel strategies to protect normal tissues from radiation injury.

## PRESENTATION AT A CONFERENCE

This study was presented at the 46^th^ annual meeting of Korean Cancer Association and the 6^th^ International Cancer Conference in Seoul, South Korea in November 2020.

## CONFLICT OF INTEREST

There is no conflict of interest to declare.
